# Susceptibility of Human Head and Neck Cancer Cells to Combined Inhibition of Glutathione and Thioredoxin Metabolism

**DOI:** 10.1371/journal.pone.0048175

**Published:** 2012-10-31

**Authors:** Arya Sobhakumari, Laurie Love-Homan, Elise V. M. Fletcher, Sean M. Martin, Arlene D. Parsons, Douglas R. Spitz, C. Michael Knudson, Andrean L. Simons

**Affiliations:** 1 Department of Pathology, The University of Iowa, Iowa City, Iowa, United States of America; 2 Interdisciplinary Human Toxicology Program, The University of Iowa, Iowa City, Iowa, United States of America; 3 Free Radical and Radiation Biology Program, Department of Radiation Oncology, The University of Iowa, Iowa City, Iowa, United States of America; 4 Holden Comprehensive Cancer Center, The University of Iowa, Iowa City, Iowa, United States of America; University of California, Merced, United States of America

## Abstract

Increased glutathione (GSH) and thioredoxin (Trx) metabolism are mechanisms that are widely implicated in resistance of cancer cells to chemotherapy. The current study determined if simultaneous inhibition of GSH and Trx metabolism enhanced cell killing of human head and neck squamous cell carcinoma (HNSCC) cells by a mechanism involving oxidative stress. Inhibition of GSH and Trx metabolism with buthionine sulfoximine (BSO) and auranofin (AUR), respectively, induced significant decreases in clonogenic survival compared to either drug alone in FaDu, Cal-27 and SCC-25 HNSCC cells *in vitro* and *in vivo* in Cal-27 xenografts. BSO+AUR significantly increased glutathione and thioredoxin oxidation and suppressed peroxiredoxin activity *in vitro*. Pre-treatment with N-acetylcysteine completely reversed BSO+AUR-induced cell killing in FaDu and Cal-27 cells, while catalase and selenium supplementation only inhibited BSO+AUR-induced cell killing in FaDu cells. BSO+AUR decreased caspase 3/7 activity in HNSCC cells and significantly reduced the viability of both Bax/Bak double knockout (DKO) and DKO-Bax reconstituted hematopoietic cells suggesting that necrosis was involved. BSO+AUR also significantly sensitized FaDu, Cal-27, SCC-25 and SQ20B cells to cell killing induced by the EGFR inhibitor Erlotinib *in vitro*. These results support the conclusion that simultaneous inhibition of GSH and Trx metabolism pathways induces oxidative stress and clonogenic killing in HNSCCs and this strategy may be useful in sensitizing HNSCCs to EGFR inhibitors.

## Introduction

Acquired resistance to chemotherapy is a major obstacle to successful head and neck squamous cell carcinoma (HNSCC) treatment. Early stage HNSCC patients have a high risk of developing secondary tumors even after local control is achieved [1]–[3], therefore, understanding the molecular mechanisms associated with chemotherapy resistance in cancer cells could lead to improvements in patient survival.

**Figure 1:**
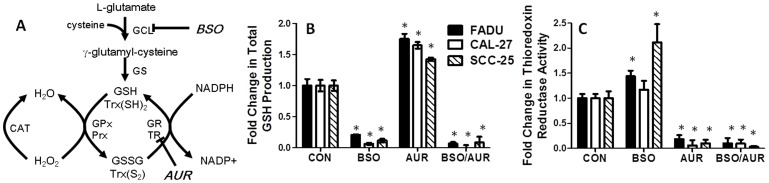
Buthionine-[S,R]-sulfoximine (BSO) and auranofin (AUR) affected glutathione and thioredoxin metabolism. A: NADPH is a source of reducing equivalents for the glutathione system consisting of reduced glutathione (GSH), glutathione disulfide (GSSG), glutathione peroxidase (GPx), and glutathione reductase (GR) and the thioredoxin system consisting of reduced thioredoxin [Trx(SH)_2_], thioredoxin disulfide [Trx(S_2_)], peroxiredoxin (Prx), and thioredoxin reductase (TR). BSO inhibits γ-glutamylcysteine ligase (GCL), which catalyzes the reaction between cysteine and L-glutamate to form γ-glutamyl-cysteine. Glutathione synthetase (GS) converts γ-GCS into GSH. AUR inhibits TR activity. FaDu, Cal-27 and SCC-25 cells were treated with 0.5 µM AUR and/or 1 mM BSO for 24 h and analyzed for total GSH (B) and TR activity (C). Error bars represent the standard error of the mean (SEM) of N = 3 experiments *, p<0.05 versus control.

Increased glutathione (GSH) and thioredoxin (Trx) metabolism are mechanisms that have been widely implicated in chemotherapy resistance [4]–[7] and both of these metabolism pathways play an important role in reactive oxygen species (ROS) detoxification [8]–[11]. The GSH system functions via glutathione peroxidase (GPx) enzymes, which inactivate H_2_O_2_ and other hydroperoxides (including alkyl and lipid peroxides) by conversion of GSH to glutathione disulfide (GSSG), which is converted back to GSH by glutathione reductase (GR) using NADPH ([12], **Figure 1A**). The Trx system is involved in the detoxification of H_2_O_2_ and hydroperoxides via the action of peroxiredoxins (Prx). During this process, oxidized Trx (Trx[S_2_]) is formed which is then reduced by thioredoxin reductase (TR) also using reducing equivalents from NADPH (**Figure 1A** [13]).

Numerous studies over the years have explored strategies of individually inhibiting GSH or Trx metabolism in addition to conventional chemotherapy agents, but have yielded variable results [14]–[16] probably due to the redundant protective functions of these systems [17]–[20]. Given that both systems detoxify H_2_O_2_ and use NADPH as reducing equivalents, it is logical that both GSH and Trx systems have overlapping and redundant functions in the detoxification of ROS. To overcome the redundancy in these pathways as they relate to resistance to therapy in HNSCC, the current study determined the effect of simultaneously inhibiting both the GSH and Trx metabolism using buthionine sulfoximine (BSO; an inhibitor of GSH synthesis), and auranofin (AUR; an inhibitor of TR activity *in vitro* and *in vivo*. This strategy was found to be very effective at enhancing oxidative stress-mediated tumor cell killing and enhancing sensitivity to Erlotinib chemotherapy.

## Materials and Methods

### Cells and culture conditions

FaDu, Cal-27 and SCC-25 human head and neck squamous carcinoma (HNSCC) cells were obtained from the American Type Culture Collection (ATCC, Manassas, VA). SQ20B HNSCC cells [21] were a gift from Dr. Anjali Gupta (Department of Radiation Oncology, The University of Iowa). All HNSCC cell lines were p53 mutant. HNEpC cells were obtained from PromoCell (Heidelberg, Germany). All cell lines were authenticated by the ATCC for viability (before freezing and after thawing), growth, morphology and isoenzymology. Cells were stored according to the supplier's instructions and used over a course of no more than 3 months after resuscitation of frozen aliquots. Bax/Bak double-knockout (DKO) and DKO-Bax reconstituted mouse hematopoietic cells were a generous gift from Dr. Craig Thompson (University of Pennsylvania, Philadelphia, PA). FaDu, Cal-27 and SQ20B cells were maintained in Dulbecco's modified Eagle's medium (DMEM) containing 4 mM L-glutamine, 1 mM sodium pyruvate, 1.5 g/L sodium bicarbonate and 4.5 g/L glucose with 10% fetal bovine serum (FBS; Hyclone, Logan, UT). SCC-25 cells were maintained in a 1∶1 mixture of Dulbecco's modified Eagle's medium and Ham's F12 medium containing 1.2 g/L sodium bicarbonate, 2.5 mM L-glutamine, 15 mM HEPES, 0.5 mM sodium pyruvate, 4.5 g/L glucose, and 400 ng/mL hydrocortisone with 10% fetal bovine serum. DKO and DKO-Bax cells were maintained in RPMI 1640 supplemented with 10% FBS, 2 mM L-glutamine, 100 U/mL penicillin, 100 g/mL streptomycin and 50 µM 2-mercaptoethanol. HNEpC cells were maintained in Airway Epithelial Growth Medium (PromoCell) containing 4 µL/mL bovine pituitary extract, 10 ng/mL epidermal growth factor, 5 µg/mL insulin, 0.5 µg/mL hydrocortisone, 0.5 µg/mL epinephrine, 6.7 ng/mL triiodo-L-thyronine and 0.1 ng/mL retinoic acid. Cultures were maintained in 5% CO_2_ and humidified in a 37°C incubator.

### Drug Treatment

Pegylated catalase (CAT), staurosporine (STS), ionomycin (ION) and L-buthionine-[S,R]-sulfoximine (BSO) were obtained from Sigma Chemical Co. (St. Louis, MO). Auranofin (AUR) was obtained from ICN Biochemicals (Aurora, OH). Erlotinib (ERL) marketed as Tarceva and N-acetylcysteine (NAC) marketed as Acetadote (Cumberland Pharmaceuticals, Nashville TN), were obtained from the inpatient pharmacy at the University of Iowa Hospitals and Clinics. All drugs were used without further purification. Drugs were added to cells at final concentrations of 1 mM BSO, 0.5 µM AUR, 20 mM NAC, 1000 U/mL CAT, 10 µM ERL, 10 µM STS and 100 µM ION. BSO, CAT, and SEL were dissolved in phosphate buffered saline (PBS). AUR, STS, ERL and ION were dissolved in dimethyl sulfoxide (DMSO). The required volume of each drug was added to cell culture media on cells to achieve the desired final concentrations. Vehicle controls were included with each experiment.

### Glutathione assay

Reduced glutathione (GSH) and glutathione disulfide (GSSG) were determined using a commercial glutathione assay kit (Cayman Chemical, Ann Arbor, MI). All glutathione determinations were normalized to the protein content of whole homogenates using the Bradford method.

### Thioredoxin Reductase Assay

Thioredoxin reductase (TR) activity was determined spectrophotometrically using a commercial thioredoxin reductase assay kit (Cayman Chemical, Ann Arbor, MI). Protein concentrations were determined by the Bradford method.

### Cell Viability

Cell viability was measured using Prestoblue™ Cell Viability reagent (Invitrogen, USA) according to the manufacturer's protocol.

### Clonogenic cell survival experiments

Clonogenic survival was determined as previously described [21]. Individual assays were performed with multiple dilutions with at least four cloning dishes per data point, repeated in at least 3 separate experiments.

### siRNA Transfection

Thioredoxin reductase (TR) and control siRNA were purchased from Santa Cruz Biotechnology (Santa Cruz, CA). HNSCC cells were transfected with 20 nM siRNA at 80% confluence in reduced-serum Eagle's Minimum Essential Medium (EMEM, Santa Cruz, CA) for 24 h. Lipofectamine 2000 (Invitrogen, Carlsbad, CA) was used for transfections following protocols provided by the manufacturer. Biochemical analyses were performed 48–72 h after transfection.

### Caspase 3/7 Activity

Caspase 3/7 activity was assessed using the ApoTox-Glo™ Triplex Assay (Promega Corporation, Madison WI).

### Thioredoxin redox western blots

Thioredoxin Western blots were performed as previously described [22]–[25]. Cells were incubated with either 2 mM dl-dithiothreitol (DTT) or 2 mM H_2_O_2_ for 10 min at room temperature, before incubation with 50 mM IAA to be used as controls to aid in the identification of thioredoxin redox state bands.

### Glutathione Reductase (GR) Assay

GR activity was measured according to the method described by Mavis and Stellwagen [26]. Data was normalized per mg protein as determined by the Lowry protein assay.

### Glutathione Peroxidase (GPx) Activity

Selenium dependent GPx activity was measured as described previously [27]. Data was normalized per mg protein as determined by the Lowry protein assay.

### Peroxiredoxin Activity Assay

2-Cys-Peroxiredoxin activity was measured as described [28]. In brief, the initial rate of NADPH oxidation was monitored spectrophotometrically at 340 nm at 30°C in a reaction mixture (150 µL) containing 50 mM Hepes-NaOH (pH 7.0), 0.25 mM NADPH, 46 nM TR, 2.4 mM Trx and 0.13 mM H_2_O_2_. The reaction was initiated by the addition of H_2_O_2_ and monitored for 10 min.

### Catalase Activity Assay

CAT activity was measured on cell homogenates by monitoring the disappearance of 10 mmol/L H_2_O_2_ in 50 mmol/L potassium phosphate (pH = 7.0) spectrophotometrically at 240 nm. Activities were expressed in mk units/mg protein as described [29].

### Tumor cell implantation

Female 4–5 week old athymic-nu/nu nude mice were purchased from Harlan Laboratories (Indianapolis, IN). Mice were housed in a pathogen-free barrier room in the Animal Care Facility at the University of Iowa and handled using aseptic procedures. All procedures were approved by the IACUC committee of the University of Iowa and conformed to the guidelines established by the NIH. Mice were allowed at least 3 days to acclimate prior to beginning experimentation, and food and water were made freely available. Tumor cells were inoculated into nude mice by subcutaneous injection of 0.1 mL aliquots of saline containing 4×10^6^ Cal-27 cells into the right flank using 26-gauge needles.

### Tumor measurements

In the *in vivo* experiments mice started drug treatment 1 week after tumor inoculation with an average tumor volume of 0.025 cm^3^. Mice were evaluated daily and tumor measurements taken three times per week using Vernier calipers. Tumor volumes were calculated using the formula: tumor volume = (length×width^2^)/2 where the length was the longest dimension, and width was the dimension perpendicular to length.

### *In vivo* drugs administration

Mice were divided into 4 groups (n = 6–10 mice/group). BSO group: BSO was dissolved in saline and administered 400 mg/kg i.p. every day for 2 weeks. AUR group: AUR stock solution was diluted with saline and administered i.p. 1 mg/kg every day for 2 weeks. BSO+AUR group: mice were administered 400 mg/kg BSO plus 1 mg/kg AUR i.p. every other day for 2 weeks. Control group: mice were administered a saline solution every day i.p. Mice were euthanized via CO_2_ gas asphyxiation or lethal overdose of sodium pentobarbital (100 mg/kg) when tumor diameter exceeded 1.5 cm in any dimension.

### Statistical Analysis

Statistical analysis was done using GraphPad Prism version 5 for Windows (GraphPad Software, San Diego, CA). Differences between 3 or more means were determined by one-way ANOVA with Tukey post-tests. Linear mixed effects regression models were used to estimate and compare the group-specific change in tumor growth curves. All statistical analysis was performed at the p<0.05 level of significance.

## Results

### BSO and AUR decreased GSH synthesis and TR activity

BSO and AUR are widely known inhibitors of cellular GSH synthesis and TR activity respectively as illustrated in the simplified schematic in **Figure 1A**. To confirm these effects of BSO and AUR in HNSCC cells, exponentially growing FaDu, Cal-27 and SCC-25 cells were treated with 1 mM BSO and/or 0.5 µM AUR for 24 h then analyzed for total GSH levels and TR activity. GSH production was significantly depleted in both BSO and BSO+AUR treated cells in all 3 cell lines, suggesting that BSO was indeed capable of inhibiting GSH synthesis (**Figure 1B**). BSO also significantly increased TR activity in FaDu and SCC-25 cells and showed a trend toward increased TR activity in Cal-27 cells (**Figure 1C**). Additionally, TR activity was inhibited in AUR and BSO+AUR treated cells confirming the mechanism of action of AUR (**Figure 1C**). AUR also increased GSH production in all 3 cell lines (**Figure 1B**). These results suggest that BSO and AUR inhibit GSH production and TR activity respectively after 24 h treatment in HNSCC cells *in vitro*.

### BSO and AUR decreased cell viability and clonogenic survival

**Figure 2:**
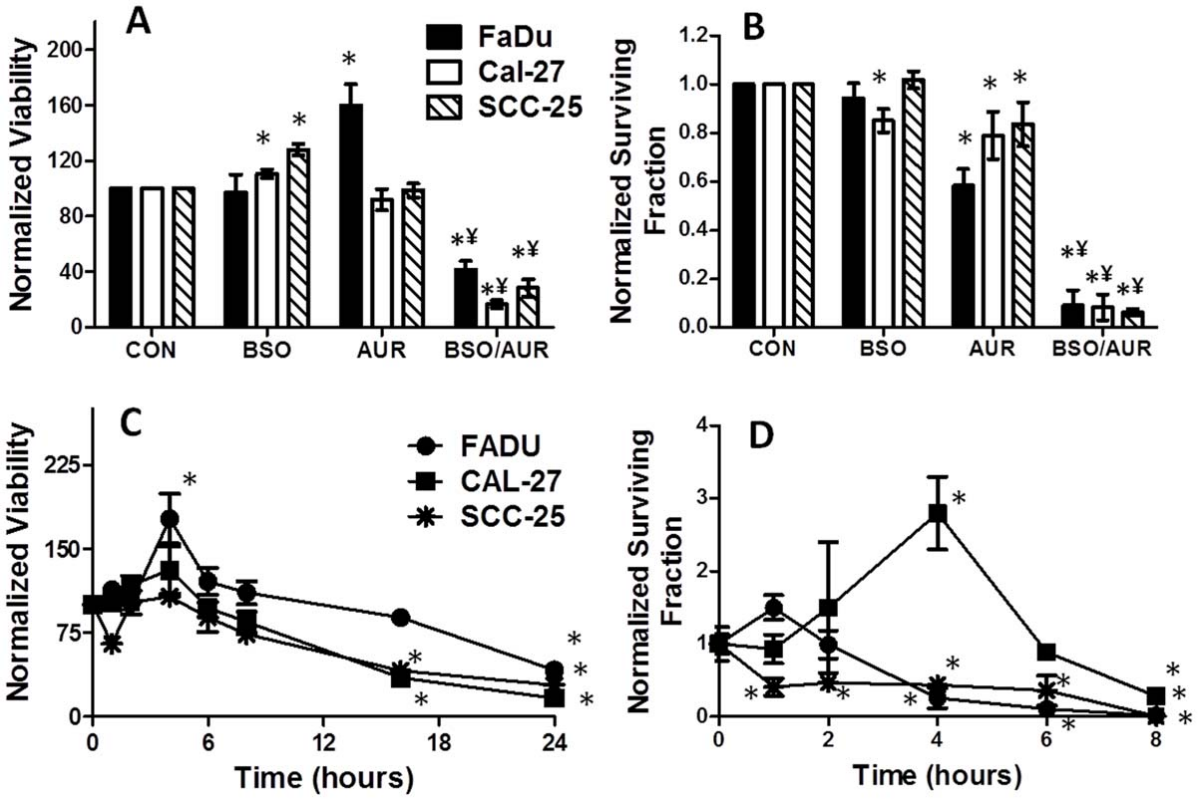
BSO and AUR decreased cell viability and clonogenic survival. FaDu, Cal-27 and SCC-25 cells were treated with 0.5 µM AUR and/or 1 mM BSO for 24 h and analyzed for cell viability (A) and clonogenic cell survival (B). Cells were treated as mentioned above and viability (C) and clonogenic cell survival (D) were measured over a period of 8 h (clonogenic survival, [D]) or 24 h (viability, [C]). Error bars represent the standard error of the mean (SEM) of N = 3 experiments. *, p<0.05 versus control; ¥, p<0.05 versus BSO or AUR.

**Figure 3:**
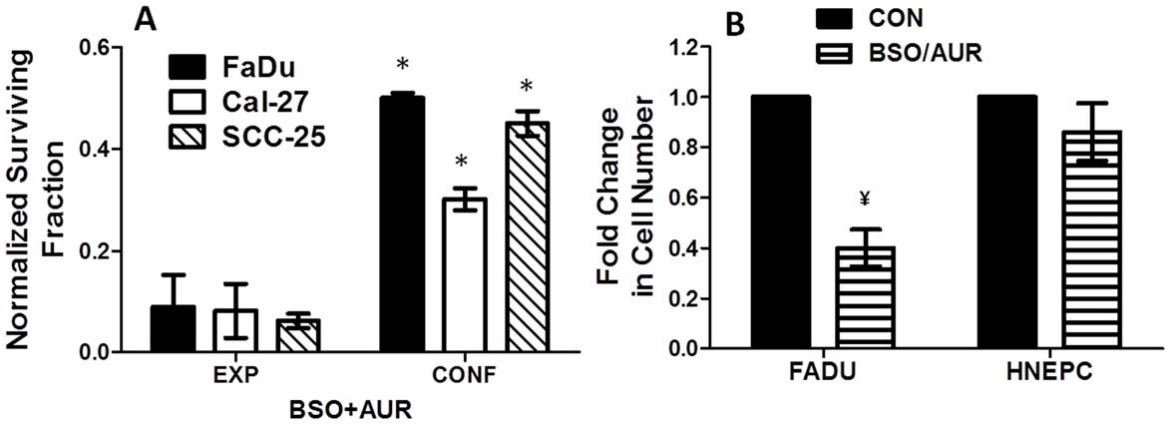
Sensitivity to BSO+AUR is decreased in confluent cancer cells and normal epithelial cells. A: Exponential growing and confluent FaDu, Cal-27 and SCC-25 cells were treated with 0.5 µM AUR and/or 1 mM BSO for 24 h and analyzed for clonogenic survival. Clonogenic cell survival data were normalized to exponentially growing and confluent control cells (not shown). B: FaDu and HNEpC cells were treated with BSO+AUR and the number of viable attached cells was counted after 24 h. Numbers of viable BSO+AUR-treated cells were normalized to their respective controls (CON). Error bars represent the standard error of the mean (SEM) of N = 3 experiments. *, p<0.05 versus EXP; ¥, p<0.05 versus CON.

To investigate the cytotoxic effects of BSO and AUR on HNSCC cells, cell viability and clonogenic survival were tested after BSO and AUR treatment in exponentially growing FaDu, Cal-27 and SCC-25 cells. BSO and AUR as single agents did not induce any significant reduction in metabolic cell viability although an increase in viability was observed with BSO treatment (in Cal-27 and SCC-25 cells) and with AUR treatment (in FaDu cells, **Figure 2A**). In contrast, the combination of BSO and AUR significantly reduced cell viability in all 3 cell lines compared to the other treatment groups (**Figure 2A**). Similarly, significant clonogenic cell killing was observed with the combination of BSO and AUR in all 3 cell lines compared to either agent alone suggesting that BSO and AUR must be used at the same time in order to induce cell killing in HNSCC cells (**Figure 2B**). When cell viability in response to BSO+AUR was tested over a 24 h period, it appeared that significant reductions in cell viability were not observed until 16 h (Cal-27 and SCC-25) and 24 h (FaDu) after treatment (**Figure 2C**). In contrast, significant reduction in clonogenic survival in response to BSO+AUR began to appear as soon as 1 h after treatment in SCC-25 cells and 4 h after treatment in FaDu cells (**Figure 2D**). These results clearly demonstrate that monitoring changes in cell viability as a function of time do not necessarily reflect drug-induced cell killing as measured by colony forming ability. We additionally observed that BSO+AUR-induced cytotoxicity measured by clonogenic assay, was significantly less in confluent HNSCC cells when compared to exponentially growing cancer cells (**Figure 3A**) suggesting that BSO+AUR was more effective in exponentially growing cells. Additionally, FaDu cells were significantly more sensitive than normal human epithelial cells (HNEpCs) to BSO+AUR after 24 h, suggesting that BSO+AUR was preferentially toxic to HNSCC cells compared to normal “untransformed” cells (**Figure 3B**). Altogether, the results in both the viability and clonogenic experiments suggest that BSO+AUR appear to induce more than additive cell killing in FaDu, Cal-27 and SCC-25 cells *in vitro*.

### Thioredoxin Reductase (TR) knockdown sensitized FaDu cells to BSO

**Figure 4:**
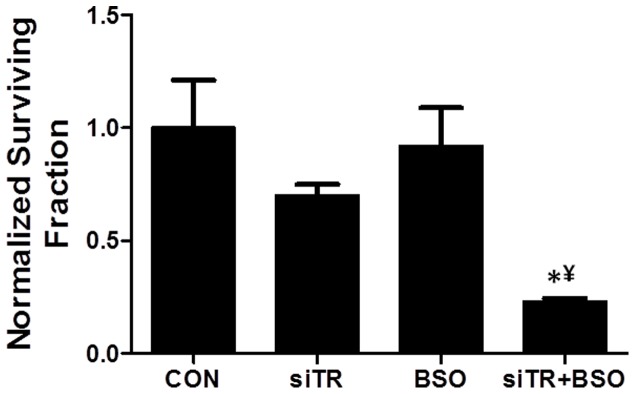
Knockdown of thioredoxin reductase (TR) increased the response to BSO in FaDu cells. TR expression was knocked down in FaDu cells using siRNA targeted to TR and analyzed for clonogenic survival with and without 1 mM BSO treatment for 24 h. Clonogenic survival data was normalized to control (CON). Error bars represent the standard error of the mean (SEM) of N = 3 experiments. *, p<0.05 versus control; ¥, p<0.05 versus BSO or siTR.

To confirm that AUR-induced changes in cytotoxicity were due to suppression of TR activity, TR expression was knocked down with siRNA targeted to TR in FaDu cells and treated with or without BSO for 24 h. TR knockdown resulted in a significant suppression of TR activity (**Table 1**) and sensitized FaDu cells to BSO as determined by clonogenic assay (**Figure 4**). These results provide further support for the hypothesis that inhibition of Trx metabolism sensitizes HNSCC cells to cell killing in the presence of inhibitors of GSH metabolism.

**Table 1:** Thioredoxin Reductase (TR) Activity in TR siRNA-treated FaDu cells.

Treatment	siCON	siTR	SiCON+BSO	SiTR+BSO
TR Activity (mU/mg protein)	1.6±0.2	0.7±0.1	1.6±0.2	0.8±0.1

### BSO+AUR induced necrotic cell death

**Figure 5:**
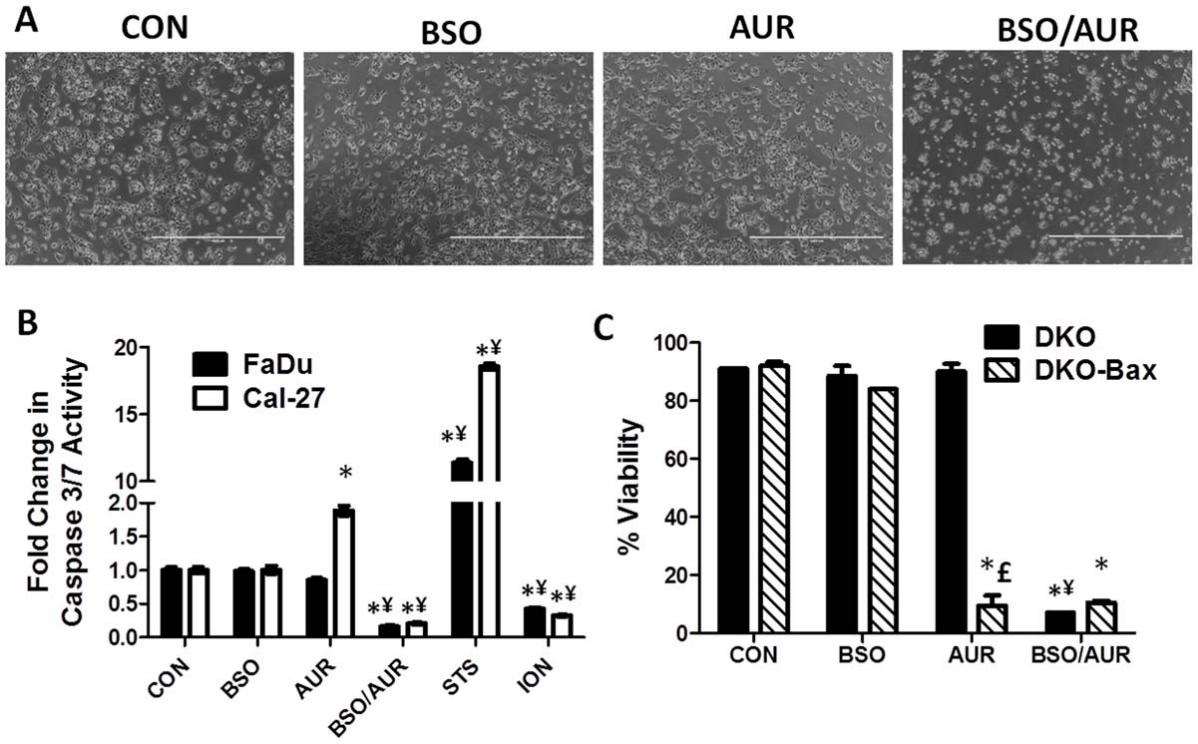
BSO+AUR induced necrotic cell death. A: Phase contrast pictures of Cal-27 cells were taken after 6 h of treatment with 0.5 µM AUR and/or 1 mM BSO. B: FaDu and Cal-27 cells were treated with 0.5 µM AUR and/or 1 mM BSO for 24 h, then analyzed for caspase 3/7 activity using a luminescence assay. Cell treatment with staurosporine (STS) and ionomycin (ION) for 6 h was used as positive controls for apoptosis and necrosis respectively. All treatments were normalized to control. *, p<0.05 versus control; ¥, p<0.05 versus BSO or AUR. C: Bax/Bak double knockout (DKO) cells and DKO cells with reconstituted Bax (DKO-Bax) were treated with 0.05 mM BSO and 2 µM AUR for 24 h then analyzed for cell viability. Error bars represent the standard error of the mean (SEM) of N = 3 experiments. *, p<0.05 versus control; ¥, p<0.05 versus BSO or AUR; £, p<0.05 versus DKO cells.

The cytotoxic response of BSO+AUR could be detected morphologically using phase contrast microscopy. Cal-27 cells treated with BSO and/or AUR for only 6 h were rounded and detached from the tissue culture dishes compared to BSO or AUR-treated cells that were attached and looked intact (**Figure 5A**). The same observations were seen in FaDu and SCC-25 cells (data not shown). To determine if apoptosis or necrosis was involved in BSO+AUR-induced cell death, we analyzed caspase 3/7 activity in response to BSO and/or AUR for 24 h in FaDu and Cal-27 cells. Cells treated with staurosporine (STS, 10 µM, 6 h) and ionomycin (ION, 100 µM, 6 h) were used as positive controls for apoptosis and necrosis respectively. We found that AUR significantly increased caspase 3/7 activity compared to control treated cells in only Cal-27 cells (**Figure 5B**). However, BSO+AUR significantly decreased caspase 3/7 activity in FaDu and Cal-27 cells, which was comparable to ionomycin treated cells (**Figure 5B**), suggesting that necrosis and not apoptosis was involved in the mechanism of cell death. In support of these results, we additionally investigated the effect of BSO+AUR on Bax^−/−^Bak^−/−^ double knock out (DKO) mouse hematopoietic cells. The apoptotic pathway is abrogated in DKO cells by genetic deletion of the pro-apoptotic factors, Bax and Bak rendering these cells dependent on necrosis when exposed to lethal insults [30]. We also used DKO cells that were reconstituted with Bax (DKO-Bax) by transfection with a vector containing Bax (pCDNA3/Bax) as previously described [30]. Reconstitution of Bax into DKO cells has been shown to restore their sensitivity to apoptotic stimuli [30], [31]. We observed that BSO alone did not affect the viability of either DKO or DKO-Bax cells (**Figure 5C**). However, DKO-Bax but not DKO cells were highly sensitive to AUR treatment (**Figure 5C**) which supports prior reports that AUR induces an apoptotic response [32]. Both DKO and DKO-Bax cells were highly sensitive to BSO+AUR suggesting that necrosis was the cell death pathway involved in response to BSO+AUR (**Figure 5C**). These results suggest that BSO+AUR at the doses and treatment times used in these studies induced necrotic cell death.

### BSO+AUR induced GSH and Trx oxidation

**Figure 6:**
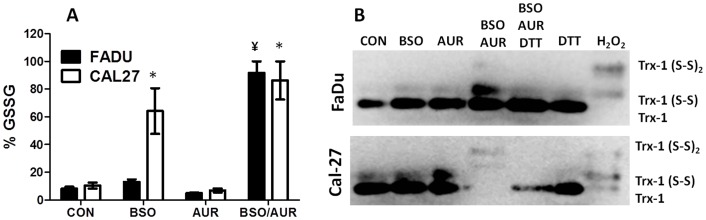
BSO+AUR induced parameters of oxidative stress. A,B: FaDu and Cal-27 cells were treated with 0.5 µM AUR and/or 1 mM BSO for 24 h, then analyzed for percentage glutathione disulfide (%GSSG, (A)) and thioredoxin redox status (B). Dithiotrietol (DTT, 2 mM) or 2 mM H_2_O_2_ was added for 15 min to control lysates as positive controls for reduced and oxidized thioredoxin respectively (B). *, p<0.05 versus control (CON); ¥, p<0.05 versus BSO or AUR.

**Figure 7:**
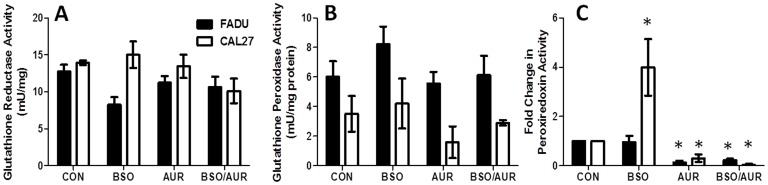
BSO+AUR affected antioxidant enzyme activity. A–C: FaDu and Cal-27 cells were treated with 0.5 µM AUR and/or 1 mM BSO for 24 h, then analyzed for glutathione reductase (GR) activity (A), glutathione peroxidase (GPx) activity (B), and peroxiredoxin activity (C). Error bars represent the standard error of the mean (SEM) of N = 3 experiments. *, p<0.05 versus control.

Because an increase in oxidized GSSG (%GSSG) is believed to signify a shift towards a more highly oxidizing intracellular environment indicative of oxidative stress [12], we investigated changes in %GSSG in response to BSO and AUR. BSO+AUR induced a significant increase in %GSSG compared to BSO and AUR alone in FaDu cells, while both BSO and BSO+AUR treated groups induced a significant increase in %GSSG compared to control in Cal-27 cells (**Figure 6A**). Analysis of thioredoxin-1 (Trx-1) redox western blot experiments showed that treatment with BSO+AUR in FaDu cells resulted in an increase in oxidized Trx-1 (Trx1[S_2_] and Trx1[S_2_]_2_) expression as seen by the increased expression of the upper 2 bands in Figure 6B compared to the other treatment groups. A similar effect was seen in Cal-27 cells in response to BSO+AUR treatment, although the total amount of Trx (reduced +oxidized) appeared to be less than the other treatment groups (**Figure 6B**). Prior reports have indicated that the reduction in total Trx expression may be due to the formation of large thioredoxin mixed protein disulfide complexes that are unable to enter the gel during electrophoresis [23]. We confirmed this by incubating BSO+AUR-treated lysates with DTT to reduce any mixed protein disulfides before analysis for reduced and oxidized Trx1. We found that DTT was successful at reducing the oxidized Trx1 formed by BSO+AUR and restoring the levels of reduced Trx1 to near control levels in both FaDu and Cal-27 cells (**Figure 6B**) suggesting that mixed protein disulfides were being formed in response to BSO+AUR. Finally, changes in the activity of other GSH and Trx related enzymes such as glutathione reductase (GR), glutathione peroxidase (GPx) and peroxiredoxin (Prx) in response to BSO and AUR were examined in FaDu and Cal-27 cells. There were no significant changes in GR (**Figure 7A**) or GPx (**Figure 7B**) in response to BSO+AUR in either cell line compared to control. Prx activity was significantly increased in BSO-treated Cal-27 cells but was significantly suppressed in AUR and BSO+AUR-treated FaDu and Cal-27 cells (**Figure 7C**). These results suggest that BSO+AUR induced oxidative stress via increased GSH and Trx oxidation in HNSCC cells.

### BSO+AUR-induced cytotoxicity is inhibited by antioxidants

**Figure 8:**
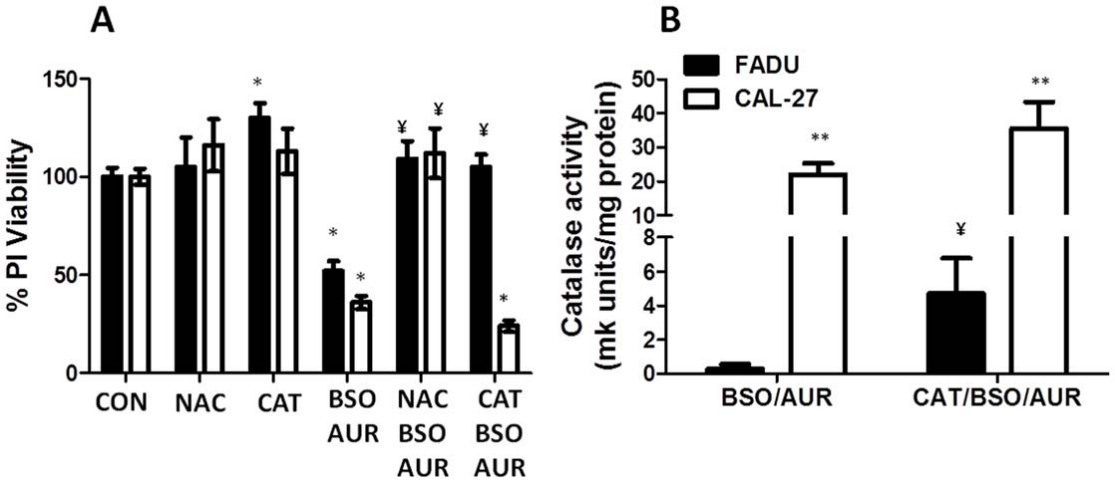
NAC and catalase protected cells from BSO+AUR. A: FaDu and Cal-27 cells were treated with 20 mM NAC or 1000 U/mL pegylated catalase (CAT) for 1 hr prior and during 24 h treatment with 0.5 µM AUR and/or 1 mM BSO. Drug treated cells were measured for cell viability. All treatments were normalized to control. B: BSO+AUR and CAT+BSO+AUR-treated FaDu and Cal-27 cells were analyzed for CAT activity. Error bars represent the standard error of the mean (SEM) of N = 3 experiments. *, p<0.05 versus control; ¥, p<0.05 versus BSO+AUR; **, p<0.05 versus respective treatment in FaDu cells.

To further analyze the role of oxidative stress in BSO+AUR-induced cell killing, FaDu and Cal-27 cells were pretreated with 20 mM NAC (a thiol antioxidant) for 1 h before and during BSO+AUR treatment, then analyzed for clonogenic survival. NAC was able to completely reverse the cytotoxicty induced by BSO+AUR in FaDu and Cal-27 cells suggesting that inhibition of BSO+AUR induces oxidative stress via disruptions in thiol metabolism (**Figure 8A**). To confirm that H_2_O_2_ was involved in BSO+AUR-induced cytotoxicity, FaDu and Cal-27 cells were pretreated for 1 h with 1000 U/mL pegylated catalase (CAT) before treatment with BSO+AUR. CAT significantly reversed BSO+AUR-induced cytotoxicity in FaDu cells but not Cal-27 cells (**Figure 8A**). Analysis of CAT activity in BSO+AUR versus CAT+BSO+AUR-treated cells revealed that treatment with CAT did not increase CAT activity in Cal-27 cells compared to FaDu cells (**Figure 8B**), suggesting that the CAT may have not adequately entered the cells. Furthermore, Cal-27 cells possessed a significantly higher level of CAT activity compared to FaDu cells (**Figure 8B**). Altogether, the results in figures 6, 7 and 8 support the hypothesis that H_2_O_2_ –induced disrutions in thiol metabolism leading to oxidative stress are involved in the cell killing induced by BSO+AUR in human HNSCC cells.

### BSO+AUR suppressed Cal-27 tumor growth

**Figure 9:**
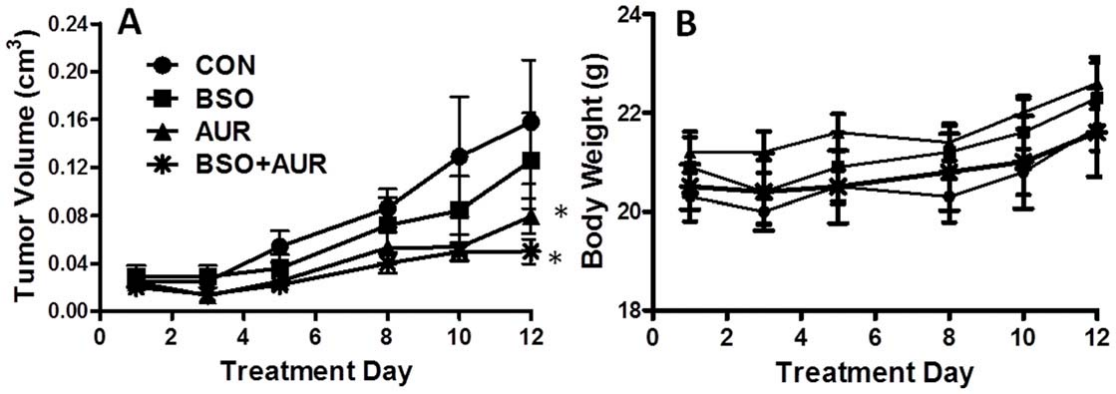
BSO+AUR suppressed Cal-27 tumor growth. A,B: Athymic (nu/nu) mice bearing Cal-27 xenograft tumors were treated beginning at an average tumor volume of 0.025 cm^3^ with 450 mg/kg BSO i.p. and/or 1 mg/kg AUR i.p. daily for 10 days. Control mice received 10% ethanol in saline i.p. daily for 10 days. Tumor volume (A) and body weight (B) was measured at day 1, 3, 5, 8, 10 and 12 of treatment. Data points represent the average values for 10 mice. B: Error bars represent the standard error of the mean (SEM) of N = 3 experiments. *, p<0.05 versus CON.

The *in vivo* activity of BSO and AUR in Cal-27 tumor bearing athymic nude mice was examined. The results showed that mice treated with 400 mg/kg BSO in combination with 1 mg/kg AUR i.p. daily for 10 days, showed a suppression of tumor growth compared to control and BSO-treated tumors (**Figure 9A**) without any adverse effects on body weight (**Figure 9B**) confirming the results seen *in vitro*. Although, BSO+AUR-treated tumors showed a trend toward slower growth compared to AUR-treated tumors, this difference did not reach significance (**Figure 9A**).

### BSO+AUR sensitized HNSCC cells to Erlotinib

**Figure 10:**
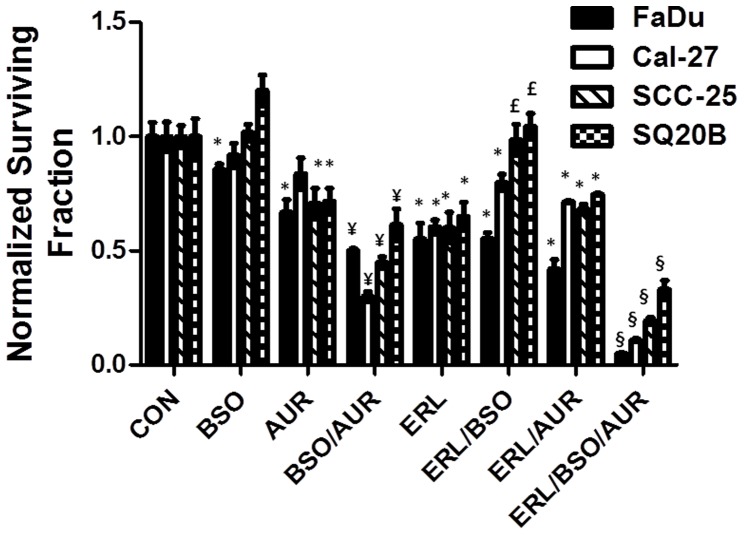
BSO+AUR sensitized HNSCC cells to Erlotinib. Confluent FaDu, Cal-27, SCC-25 and SQ20B cells were treated with 1 mM BSO and or 0.5 µM AUR in combination with 10 µM Erlotinib (ERL) for 24 h. Clonogenic cell survival data were normalized to control (CON) cells. Error bars represent the standard error of the mean (SEM) of N = 3 experiments. *, p<0.05 versus CON; ¥, p<0.05 versus CON, BSO and AUR; £, p<0.05 versus ERL; §, p<0.05 versus all other treatment groups.

Given that resistance to chemotherapy agents, such as EGFR inhibitors, is a significant limitation in HNSCC treatment [33], we determined if BSO+AUR would sensitize confluent HNSCC cells to the EGFR inhibitor Erlotinib. We found that BSO and AUR when used alone were not able to sensitize cells to Erlotinib (10 µM, 24 h (**Figure 10**)). However, BSO+AUR significantly sensitized all cell lines tested to Erlotinib (**Figure 10**) suggesting that BSO+AUR must be used in combination with EGFR inhibitors to achieve maximal chemo-sensitization.

## Discussion

Increased GSH and Trx metabolism have been known for years to be correlated with high tumor aggression and resistance to chemotherapy [4]–[7]. As a result of this knowledge, inhibition of GSH or Trx in conjunction with chemotherapy has been extensively explored but is highly cell line specific and has yielded disappointing results which may be due to the overlapping and redundant antioxidant functions of the GSH and Trx systems [17]–[20]. In fact, our prior studies have shown that the ability of BSO or AUR to sensitize HNSCC cells to Akt inhibitors was highly cell line specific [16]. Both the GSH and Trx metabolic pathways reduce H_2_O_2_ and organic hydroperoxides (including lipid and alkyl peroxides) using electrons derived from NADPH (**Figure 1A** [12], [13]). Therefore, it is likely that inhibition of GSH metabolism results in a compensatory upregulation of Trx metabolism and vice versa, which may be the reason inhibiting only one antioxidant system fails as an effective strategy to enhance cancer therapy. Our data provides several lines of evidence in support of this argument: (1) Inhibition of TR activity with AUR was able to increase GSH production in all HNSCC cell lines tested (**Figure 1B**), (2) inhibition of GSH production with BSO appeared to increase TR activity in the same cell lines (**Figure 1C**) and (3) BSO or AUR as single agents could not sensitize HNSCC cells to ERL, whereas BSO+AUR clearly induced sensitization to ERL (**Figure 10**). Because the GSH and Trx antioxidant systems appear to compensate for each other, the results of this study support our hypothesis that both antioxidant systems must be simultaneously inhibited in order to reduce H_2_O_2_ detoxification resulting in severe oxidative stress and cytotoxicity in human HNSCC cells.

Our studies indicate that using BSO+AUR to simultaneously inhibit GSH production and TR activity worked remarkably well in this HNSCC cell model. BSO is commonly used as an inhibitor of GSH synthesis by inhibiting the rate limiting enzyme in GSH synthesis (glutamate cysteine ligase; GCL), **Figure 1A**, [34]). AUR, which is a relatively specific inhibitor of TR, belongs to the gold(I)-based drug class utilized in the treatment of rheumatoid arthritis [35] and has been shown to stimulate the mitochondrial production of hydrogen peroxide [36]. AUR is believed to bind to the active site selenocysteine of TR resulting in inhibition of TR activity [37]. Both of these agents have already been tested and used safely as single agents in humans [38]–[46] but never tested in combination. Here we show that the combination of these agents induced significant cell killing in HNSCC cells *in vitro* (**Figure 2**) and *in vivo* (**Figure 9A**), and this cell killing could be detected as soon as 1 h after treatment (**Figure 2D**). In addition, knockdown of TR with siRNA targeted to TR was as effective as AUR in sensitizing cells to BSO, suggesting that the effects of AUR were due to inhibition of TR activity as expected (**Figure 4** **,** **Table 1**). However, the profound inhibition of Prx activity with AUR treatment (**Figure 7C**) was unexpected. This finding raises the possibility that suppression of TR activity may affect Prx activity since oxidized Prxs are dependent on the Trx system for recycling to their reduced forms [28]. Prior studies have shown that Prxs could be rapidly oxidized and inactivated by AUR via increased mitochondrial H_2_O_2_ or by impaired Trx metabolism [32], [47], [48]. It is also possible that AUR may directly bind to Prxs since Prxs possess a highly reactive cysteine residue in their active site. We are unable to fully decipher the mechanism of action of AUR on the suppression of Prx activity with our results so far, but this interesting observation warrants further study.

The mechanism of action of BSO+AUR-induced cell killing appears to involve oxidative stress since both antioxidant systems participate in H_2_O_2_ detoxification (**Figure 1A**). Oxidative stress parameters such as increased %GSSG and oxidized Trx were observed suggesting that BSO+AUR was indeed inducing oxidative stress (**Figure 6**). Furthermore, NAC completely reversed the cytotoxicity induced by BSO+AUR in FaDu and Cal-27 cells strongly supporting the hypothesis that disruptions in thiol metabolism were causally involved in cancer cell killing (**Figure 8A**). The role of H_2_O_2_-induced oxidative stress in BSO+AUR-induced cytotoxicity was confirmed by the rescue of BSO+AUR-induced cell killing with CAT (**Figure 8A**). High levels of CAT (1000 U/mL) were able to rescue BSO+AUR-induced cell killing in FaDu cells but not Cal-27 cells (**Figure 8A**). Given the lack of increased CAT activity in CAT+BSO+AUR-treated cells versus BSO+AUR-treated cells (**Figure 8B**), it is possible that CAT uptake was suppressed in CAL-27 cells compared to FaDu cells.

Both BSO and AUR have been shown in prior reports to induce apoptosis [32], [49]. However, the results in Figures 5B and 5C suggest that necrosis was involved in the toxicity of the combined treatment. AUR significantly increased caspase 3/7 activity in Cal-27 cells compared to control (**Figure 5B**) and suppressed the viability of DKO-Bax cells but not DKO cells (**Figure 5C**) which supports prior reports and strongly suggests the role of apoptosis in AUR-induced cell killing [32]. However, BSO+AUR significantly decreased caspase 3/7 activity (**Figure 5B**), and significantly decreased the viability of both DKO and DKO-Bax cells (**Figure 5C**), which points to necrotic cell death since DKO cells are unable to undergo apoptosis. Additionally the decrease in caspase 3/7 activity induced by BSO+AUR was comparable to ionomycin which was the positive control for necrotic cell death (**Figure 5B**). BSO+AUR-induced oxidative stress may have led to either: (1) inactivation of caspases due to oxidation of their thiol group in their active site, (2) a drastic drop in ATP levels or (3) mitochondrial dysfunction, all of which can lead to necrosis [50]. Although we do not know which mechanism is responsible for the induction of necrosis with BSO+AUR treatment, our results point to necrosis and not apoptosis as the method of BSO+AUR-cell death in our HNSCC cancer cell model at the doses and treatment times described in this study.

Although we have shown that treatment of BSO+AUR was effective and tolerated in xenograft-bearing mice (Figure 9A,B), it is still possible that this treatment would leave all cells (normal and tumor) susceptible to other mild stressors so that BSO+AUR could not be incorporated into a therapeutic regimen. However, recent studies by Fath et al. 2011, have shown that BSO+AUR could successfully be combined with carboplatin to treat lung cancer tumors in mice with no apparent signs of toxicity [25]. This suggests that BSO+AUR could be investigated for use with other common chemotherapy agents. To begin to investigate if BSO+AUR could be used as a therapeutic adjuvant in HNSCC, we determined if BSO+AUR would sensitize HNSCC cells to the EGFR inhibitor Erlotinib (ERL). EGFR signaling pathways are upregulated in the majority of HNSCC tumors and are associated with a poor clinical prognosis as these cancers express an aggressive phenotype compared to EGFR negative cancers [51], [52]. EGFR inhibitors have been incorporated into the standard management of HNSCC, but the problem of acquired drug resistance represents a barrier to long term patient survival [33]. We observed that BSO+AUR was able to significantly sensitize confluent FaDu, Cal-27, SCC-25 and SQ20B cells to Erlotinib *in vitro* (**Figure 10**). FaDu, Cal-27 and SCC-25 cells all overexpress wildtype EGFR, and we included the SQ20B cell line because it expresses a constitutively active mutation in EGFR [53]. Importantly, we observed that neither BSO nor AUR used as agents alone could sensitize cells to ERL, but both needed to be used simultaneously to achieve the desired effect (**Figure 10**). Given the success of these results, studies are now underway to determine if BSO+AUR could sensitize HNSCC cells to ERL and to other EGFR inhibitors *in vivo*.

Overall, these studies show that the simultaneously inhibiting GSH and Trx metabolism induces extreme oxidative stress and HNSCC cell killing, and this simple strategy is effective in the presence or absence of EGFR inhibitors. This strategy represents a potentially efficient way to enhance conventional chemo/radiotherapy regimens in HNSCC and appears to warrant further investigation.
